# Mitochondrial immunometabolism in sepsis: bridging immune cell dysfunction and organ failure

**DOI:** 10.3389/fimmu.2026.1921226

**Published:** 2026-08-21

**Authors:** Xingzhan Zhang, Ling Zhao, Wei Fu, Lili Pan, Huanhuan Wu, Jie Peng

**Affiliations:** 1 Department of Intensive Care Unit, The People’s Hospital Medical Group of Xiangzhou, Zhuhai, Guangdong, China; 2 Department of Intensive Care Unit, Guangzhou First People’s Hospital, School of Medicine, South China University of Technology, Guangzhou, Guangdong, China; 3 Department of Critical Care Medicine, Zhujiang Hospital, Southern Medical University, Guangzhou, Guangdong, China

**Keywords:** immune cell dysfunction, immunometabolism, metabolic reprogramming, mitochondria, mitochondrial DNA, multiple organ failure, NLRP3 inflammasome, sepsis

## Abstract

Sepsis, defined as life-threatening organ dysfunction caused by a dysregulated host response to infection, remains a leading cause of mortality in critical care, and sepsis-associated multiple organ failure continues to defy effective therapy. Increasing evidence positions mitochondria at the interface of cellular bioenergetics and innate immune signaling, making mitochondrial immunometabolism a compelling framework for understanding sepsis pathophysiology. In this mini-review, we synthesize how mitochondrial bioenergetic dysfunction shapes immune cell function across the dynamic course of sepsis, from the glycolytic, oxidative phosphorylation (OXPHOS)-uncoupled state of the hyperinflammatory phase to the bioenergetic failure of the immunoparalytic phase. We examine the contested roles of mitochondrial quality-control mechanisms: mitophagy, dynamics, and biogenesis, in immune cell remodeling, and propose that their net effect follows a time- and cell-type-dependent pattern rather than a fixed protective or deleterious role. We further discuss how mitochondrial damage-associated molecular patterns (mtDAMPs), mitochondrial DNA (mtDNA), reactive oxygen species (mtROS), and remodeled cardiolipin activate the cGAS-STING pathway and the NLRP3 inflammasome and cross-regulate one another to amplify inflammation and drive organ injury. Integrating these themes, we highlight mitochondrial immunometabolic crosstalk between key immune cell subsets (macrophages, neutrophils, and lymphocytes) and the parenchymal cells of vulnerable target organs (heart, kidney, lung, and the gut–liver axis). Finally, we identify knowledge gaps spanning temporal dynamics, cellular heterogeneity, and clinical translation, acknowledge the limitations of the current evidence, and outline emerging therapeutic and monitoring strategies. Collectively, mitochondrial immunometabolism links immune cell dysfunction to organ failure and may guide stage- and endotype-specific interventions in sepsis.

## 1 Introduction

Sepsis is life-threatening organ dysfunction caused by a dysregulated host response to infection, and its persistently high morbidity and mortality make it a major challenge in critical care medicine (1, 2). Despite advances in anti-infective therapy and organ support, sepsis-associated multiple organ failure remains the leading cause of death, and its pathophysiology is incompletely understood (2, 3). Research focus has shifted from inflammation and hemodynamic disturbance alone toward the reciprocal regulation between cellular energy metabolism and immune function. As both the central hub of energy metabolism and a key platform for innate immune signaling, mitochondria have attracted increasing attention in sepsis pathogenesis (3, 4).

Mitochondria play a dual role in sepsis. As the cell’s powerhouse, they generate ATP through OXPHOS to sustain cellular function; simultaneously, they serve as a platform for immune signaling, and the ROS and mtDNA they release activate inflammatory pathways and modulate immune cell function (5, 6). In sepsis, mitochondrial dysfunction manifests as bioenergetic failure, dynamic imbalance, disrupted quality control, and mtDAMP release; these alterations compromise the energy supply of parenchymal cells and reshape the metabolic programs and functional states of immune cells (7, 8). Immune cell dysfunction is central to sepsis evolution: early hyperinflammation and later immunosuppression occur successively or coexist (9, 10). Macrophages, neutrophils, and T cells undergo profound metabolic reprogramming, and their mitochondrial state directly determines polarization and effector function (4, 7). Concurrently, mtDNA, mtROS, and other molecules from damaged mitochondria activate cGAS-STING and the NLRP3 inflammasome, amplifying inflammation and promoting organ injury (11–13). Mitochondrial immunometabolism thus constitutes a critical bridge linking immune cell dysfunction to multiple organ failure.

**Figure 1:**
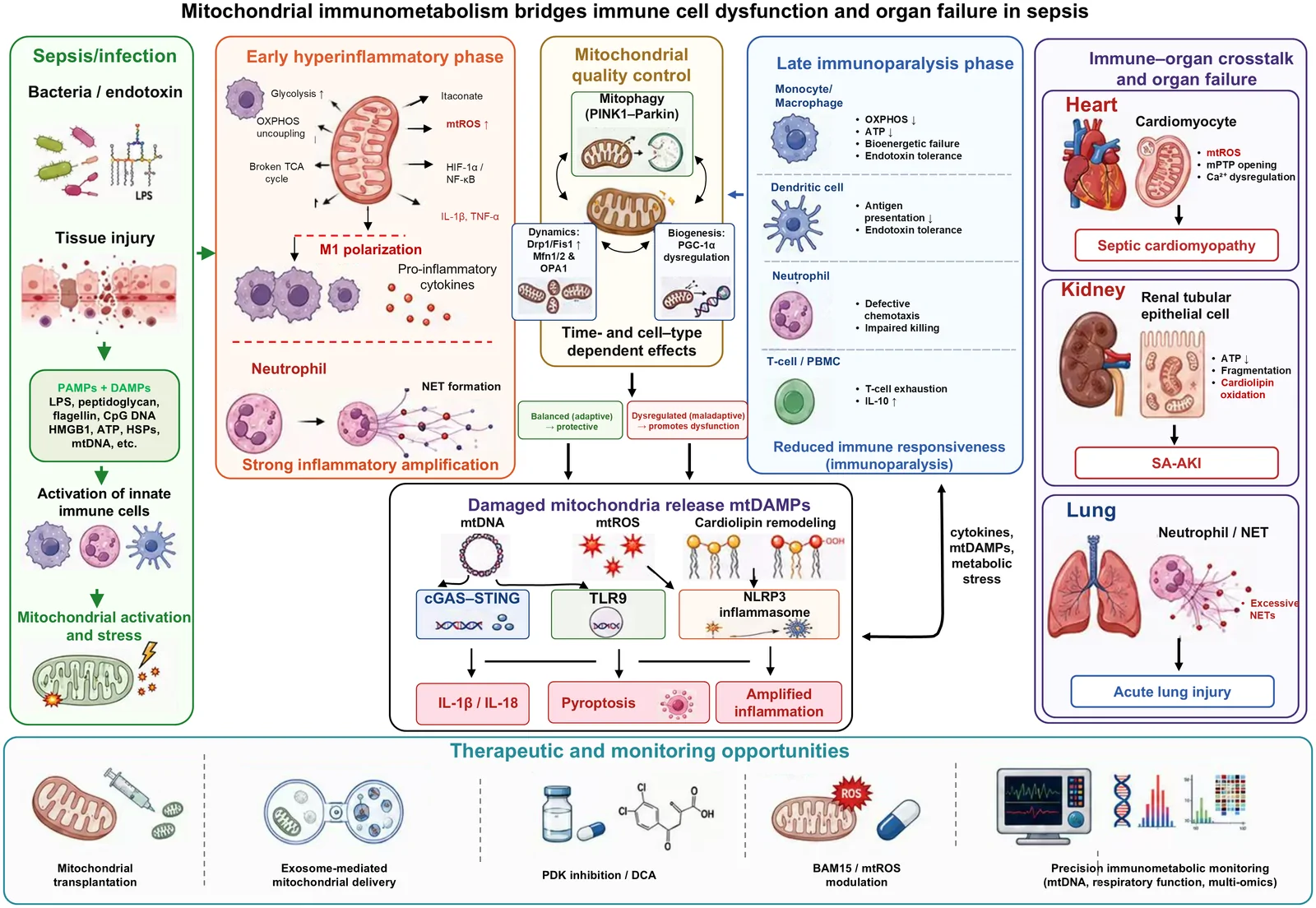
Mitochondrial immunometabolism bridges immune-cell dysfunction and organ failure in sepsis. Infection-derived PAMPs and tissue injury-associated DAMPs activate innate immune cells and induce mitochondrial activation and stress. During early sepsis, increased glycolysis, OXPHOS uncoupling, a broken TCA cycle, mtROS generation, and HIF-1α/NF-κB signaling promote pro-inflammatory cytokine production, M1 macrophage polarization, NET formation, and inflammatory amplification. As sepsis progresses, OXPHOS inhibition, ATP depletion, bioenergetic failure, endotoxin tolerance, impaired antigen presentation, defective neutrophil function, and T-cell exhaustion contribute to immunoparalysis. Dysregulated mitochondrial quality control—mitophagy, dynamics, and biogenesis—promotes release of mtDAMPs (mtDNA, mtROS, and remodeled cardiolipin), which activates cGAS-STING, TLR9, and NLRP3 inflammasome signaling. Mitochondrial immunometabolic crosstalk between immune cells and parenchymal cells contributes to septic cardiomyopathy, sepsis-associated acute kidney injury, and acute lung injury. Potential intervention and monitoring strategies include mitochondrial transplantation, exosome-mediated mitochondrial delivery, PDK inhibition/DCA, BAM15-mediated mtROS modulation, and precision immunometabolic monitoring.

This mini-review summarizes advances in mitochondrial immunometabolism in sepsis, focusing on how bioenergetic dysfunction drives immune cell dysfunction, the role of mtDAMPs in organ failure, and the immunometabolic crosstalk between immune cell subsets and target organs. We give particular attention to lymphocyte subsets, the cross-regulation of mtDAMP-sensing pathways, and the gut–liver axis, and we explicitly delineate the limitations of the current evidence. On this basis, we discuss knowledge gaps and future directions (**Figure 1**).

## 2 Literature search

This narrative mini-review was informed by a structured search of PubMed, Web of Science, and Scopus for English-language publications, emphasizing work from 2020 onward. Search terms combined two conceptual domains: the disease context (e.g., “sepsis”, “septic shock”, “sepsis-associated acute kidney injury”, “septic cardiomyopathy”) and mitochondrial/immunometabolic processes (e.g., “mitochondria”, “immunometabolism”, “metabolic reprogramming”, “OXPHOS”, “glycolysis”, “mitophagy”, “mitochondrial dynamics”, “mtDNA”, “cGAS-STING”, “NLRP3 inflammasome”, “mtROS”, “cardiolipin”, “macrophage polarization”, “neutrophil extracellular traps”). Titles and abstracts were screened, full texts of pertinent studies retrieved, and reference lists examined manually. Source selection prioritized conceptual representativeness, mechanistic relevance, and recency rather than exhaustive coverage.

The composite strategy combined three modules with Boolean operators: (Module 1, disease) (“sepsis” OR “septic shock” OR “sepsis-associated acute kidney injury” OR “septic cardiomyopathy” OR “acute lung injury” OR “sepsis-induced liver injury” OR “intestinal barrier dysfunction”) AND (Module 2, mitochondria) (“mitochondria” OR “mitophagy” OR “mitochondrial dynamics” OR “mitochondrial DNA” OR “cardiolipin” OR “oxidative phosphorylation” OR “mitochondrial reactive oxygen species”) AND (Module 3, immunometabolism) (“immunometabolism” OR “metabolic reprogramming” OR “macrophage polarization” OR “regulatory T cell” OR “NLRP3 inflammasome” OR “cGAS-STING”). Equivalent field tags were applied across databases and adapted to each interface.

## 3 Mitochondrial bioenergetic dysfunction drives immune cell dysfunction

### 3.1 Metabolic features of the hyperinflammatory phase: glycolysis and OXPHOS uncoupling

In early sepsis, pathogen- and damage-associated molecular patterns (PAMPs and DAMPs) activate innate immune cells and trigger intense inflammation. Immune cells undergo marked metabolic reprogramming, the hallmark of which is a shift from OXPHOS to aerobic glycolysis (the “Warburg effect”) (3, 10). Upon lipopolysaccharide (LPS) stimulation, macrophages and neutrophils upregulate glycolytic enzymes to rapidly generate ATP and meet the biosynthetic demands of inflammatory effector functions (3, 9). This switch also promotes, via accumulation of intermediates such as succinate, the stabilization of hypoxia-inducible factor-1α (HIF-1α) and proinflammatory cytokine production (14, 15).

Mitochondria are not quiescent but functionally uncoupled during this phase. In LPS-stimulated macrophages, OXPHOS is inhibited while membrane potential is partially maintained and mtROS production rises (4, 16). As a signaling molecule, mtROS activate nuclear factor-κB (NF-κB) and the NLRP3 inflammasome, amplifying inflammation (13, 17). The tricarboxylic acid (TCA) cycle becomes “broken”, with succinate accumulation and increased itaconate, the latter exerting anti-inflammatory, antioxidant effects by inhibiting succinate dehydrogenase, a metabolic negative-feedback mechanism (18). Activation of pyruvate dehydrogenase kinase (PDK) phosphorylates and inactivates the pyruvate dehydrogenase complex (PDC), blocking pyruvate entry into the TCA cycle and aggravating OXPHOS inhibition (19, 20); the resulting lactate accumulation regulates inflammatory gene expression through histone lactylation (21, 22).

### 3.2 Metabolic features of the immunoparalysis phase: OXPHOS inhibition and bioenergetic failure

As sepsis progresses, some patients transition from hyperinflammation to immunoparalysis, characterized by reduced immune-cell responsiveness, decreased proinflammatory cytokine production, impaired antigen presentation, and lymphocyte exhaustion (9, 23). The core mitochondrial feature is bioenergetic failure, a sustained decline in ATP synthesis that fails to meet immune-cell demands (5, 24). Mitochondrial respiratory function in peripheral blood mononuclear cells (PBMCs) of septic patients is markedly reduced and closely associated with the immunoparalytic phenotype (23). Under sustained stimulation, monocytes/macrophages develop “endotoxin tolerance”, with suppressed bioenergetics and increased anti-inflammatory interleukin-10 (16, 25).

Lymphocytes are equally shaped by mitochondrial metabolism, a dimension that deserves explicit emphasis. In late sepsis, T cells exhibit reduced mitochondrial mass and impaired oxidative metabolism, promoting exhaustion and an immunosuppressive state (26). Regulatory T cells (Tregs) display a distinctive program: whereas effector T cells favor glycolysis, Foxp3 drives Tregs toward OXPHOS and fatty acid oxidation, and in sepsis this reprogramming expands the suppressive Treg compartment and reinforces immunosuppression (27). Dendritic cells are also affected—mtDNA released during sepsis activates STING signaling, downregulating costimulatory molecules and impairing antigen presentation to sustain immunoparalysis (11). Mitochondrial bioenergetics therefore couples the innate and adaptive arms of the septic immune response.

### 3.3 The controversial role of mitochondrial quality control in immune cell remodeling

Mitochondrial quality control, mitophagy, dynamics (fission/fusion), and biogenesis maintain network homeostasis (8, 28), yet its role in sepsis is controversial. For mitophagy, some studies show protection: early clearance of damaged mitochondria limits mtROS and mtDNA release, attenuating injury (28, 29), and PINK1-Parkin mitophagy is protective in septic acute kidney injury, whereas macrophage migration inhibitory factor aggravates tubular injury by disrupting PINK1-Parkin (30). Others show the opposite—LPS plus interferon-γ inhibits PINK1-dependent mitophagy via a STAT1 pathway to promote macrophage antibacterial defense, so that inhibiting mitophagy is protective (31). Similarly, the fission protein Drp1 is upregulated in sepsis, and Drp1/Fis1-mediated pathological fission drives macrophage dysfunction and immunoparalysis (16, 32), while downregulated Mfn1/2 and OPA1 weaken network connectivity (33). PGC-1α–driven biogenesis is generally suppressed and cannot compensate for mitochondrial loss (8), yet excessive early biogenesis may generate structurally abnormal mitochondria that aggravate oxidative stress (8).

These apparently conflicting data are best reconciled by a unified, time- and cell-type-dependent regulatory model. In the early hyperinflammatory phase, moderate mitophagy and adaptive fission are protective, containing mtDAMP release and supporting antibacterial effector function; in the late immunoparalytic phase, insufficient or excessive mitophagy, unopposed Drp1/Fis1 fission, and failed PGC-1α biogenesis become deleterious, accumulating damaged mitochondria and deepening bioenergetic failure. The same mechanism can therefore be protective or injurious depending on disease stage and on the immune or parenchymal cell type involved. Accordingly, quality-control–directed therapy should be stage- and cell-specific rather than uniformly activating or inhibiting a given pathway.

## 4 The role of mitochondrial damage-associated molecular patterns in organ failure

### 4.1 Mitochondrial DNA release and activation of the cGAS-STING pathway

Mitochondria are of endosymbiotic origin and retain bacterial features; their DNA contains unmethylated CpG motifs recognized as non-self by innate immunity (12, 34). In sepsis, increased membrane permeability or mitochondrial rupture releases mtDNA into the cytoplasm and extracellular space (11, 12). Cytoplasmic mtDNA is sensed by cGAS, which synthesizes cGAMP to activate STING and initiate interferon and inflammatory gene expression (11, 33).

This pathway drives organ injury. mtDNA mediates dendritic-cell immunoparalysis by suppressing costimulatory molecules and impairing antigen presentation (11); in the kidney, it activates cGAS-STING in sepsis-associated acute kidney injury (33, 35); and circulating mtDNA acts as a DAMP activating neutrophils and macrophages via Toll-like receptor 9 to promote systemic inflammation (5, 35). mtDNA release is closely tied to quality control; unrepaired damaged mitochondria are prone to rupture, so mitophagy integrity limits mtDNA-mediated inflammation (28, 29). Mitochondrial uncouplers such as BAM15 reduce mtROS and mtDNA release, attenuating renal injury and mortality in septic mice (36).

### 4.2 The dual role of mtROS in inflammasome activation and tissue injury

mtROS are respiratory-chain by-products, markedly increased in sepsis (4, 5), with dual roles: moderate levels support antibacterial defense and signaling, whereas excess causes oxidative stress, mitochondrial damage, and tissue injury (6, 13). mtROS is a key activator of the NLRP3 inflammasome, driving caspase-1 activation, IL-1β/IL-18 maturation, and pyroptosis (13), which is important in septic myocardial dysfunction and acute kidney injury (21, 37, 38). Yet mitochondria-targeted antioxidants attenuate excessive NLRP3 activation, indicating a threshold effect (6, 39).

This threshold is set by endogenous buffering systems, which explains why antioxidant therapy cannot, and should not, abolish mtROS entirely. Uncoupling protein 2 (UCP2) produces mild uncoupling that lowers membrane potential and curbs mtROS generation, thereby restraining glycolytic and inflammatory reprogramming (25), while the mitochondrial glutathione (GSH)/glutathione peroxidase system scavenges peroxides and, once depleted, licenses lipid peroxidation, ferroptosis, and inflammasome activation (39). Because a basal mtROS tone is required for pathogen killing and redox signaling, complete pharmacological suppression risks impairing host defense; the therapeutic goal is to restore the UCP2- and GSH-defined homeostatic set-point rather than to eliminate mtROS.

The role of mtROS in tissue injury is likewise dual. Excess mtROS causes cardiomyocyte mitochondrial dysfunction, disturbed calcium homeostasis, and apoptosis in septic cardiomyopathy (37, 40), and promotes cytochrome c release by oxidizing cardiolipin (41). Conversely, neutrophils depend on the respiratory burst for pathogen killing (9, 42), so complete mtROS elimination may impair host defense, reinforcing that strategies should restore homeostasis rather than achieve total suppression.

### 4.3 Mitochondrial lipid remodeling (cardiolipin) as a hub of metabolic and immune signaling

Cardiolipin is a signature inner-membrane phospholipid essential for respiratory-chain supercomplex stability, cristae structure, and OXPHOS efficiency (41). Under septic conditions, its composition and distribution change markedly (lipid remodeling) (41). Oxidative stress peroxidizes cardiolipin, disrupting respiratory-chain interactions and impairing ATP synthesis (34, 41); cardiolipin also externalizes to the outer membrane, where it serves as a signaling platform, interacts directly with NLRP3 components, and, once oxidized and released, acts as an mtDAMP (34, 41). Impaired fatty acid oxidation (FAO) and dysregulated peroxisome proliferator-activated receptor (PPAR) signaling further aggravate dysfunction; PPARγ agonists may improve outcomes but show heterogeneous efficacy, possibly owing to patient heterogeneity (10, 15).

Considered across organs, cardiolipin oxidation and outer-membrane externalization (“phospholipid oxidative eversion”) represent a shared, stereotyped pathological effect in parenchymal injury: in cardiomyocytes, renal tubular epithelial cells, and hepatocytes alike, eversion simultaneously destabilizes the respiratory chain (a bioenergetic hit) and exposes a pro-inflammatory lipid platform (an immune hit), thereby coupling metabolic failure to inflammasome activation through a single structural lesion (41).

### 4.4 Cross-regulation of mtDAMP pathways

The mtDAMP-sensing pathways described above do not operate in isolation but form an interconnected, self-amplifying network. Damaged mitochondria release mtDNA, mtROS, and oxidized cardiolipin together, and these signals converge on and reinforce one another: mtROS primes NLRP3 while promoting further mtDNA oxidation and release; oxidized mtDNA is a particularly potent NLRP3 ligand that links the cGAS-STING and inflammasome axes; externalized cardiolipin provides the membrane platform that lowers the NLRP3 activation threshold; and type I interferon downstream of STING sensitizes cells to subsequent inflammasome signaling. The net result is feed-forward amplification in which each pathway both triggers and is triggered by the others, so that partial blockade of any single node is often insufficient. This cross-regulatory network is depicted in **Supplementary Figure 1**, and its nodes represent candidate points for combination-targeted intervention.

## 5 Mitochondrial immunometabolism of key immune cell subsets and their organ-specific interactions

### 5.1 Macrophage polarization and sepsis-associated organ injury

Macrophages are central to the septic immune response, and their polarization is tied to mitochondrial status (4, 7). Classically activated M1 macrophages rely on glycolysis with a broken TCA cycle and succinate-driven HIF-1α stabilization and IL-1β production (3, 4); alternatively activated M2 macrophages depend on OXPHOS and FAO with intact mitochondria supporting anti-inflammatory, reparative functions (4, 43). In sepsis, polarization imbalance drives organ injury—excess M1 causes cytokine storm and tissue damage, whereas excess M2 associates with immunosuppression (4, 7). Increased mtROS, reduced membrane potential, and impaired mitophagy promote M1 over M2 (7, 30), while NRF1 limits mitochondrial stress and inflammation by promoting protein turnover via the ubiquitin-proteasome system (44).

Metabolic intermediates act as polarization signals: itaconate favors an anti-inflammatory phenotype via succinate dehydrogenase inhibition and NRF2 activation (18), whereas succinate promotes proinflammatory genes via HIF-1α (3). In organ injury, macrophage mitochondrial state shapes tissue damage—renal macrophage dysfunction amplifies tubular injury in sepsis-associated acute kidney injury (33, 45), and cardiac macrophage reprogramming participates in myocardial inflammation and dysfunction (17, 32). Strategies targeting macrophage mitochondria, including mitochondrial transplantation and metabolic substrate intervention, ameliorate organ injury in animal models (46, 47).

### 5.2 Mitochondrial regulation of neutrophil function and NET formation

Neutrophils are the first line of innate defense, and although they rely mainly on glycolysis, mitochondria regulate survival, ROS production, and neutrophil extracellular trap (NET) formation (9, 42). In sepsis, decreased membrane potential and aberrant mtROS impair chemotaxis and apoptosis (16, 42), and Drp1/Fis1-mediated fragmentation releases damaged mitochondria that act as DAMPs, inducing cross-tolerance in other immune cells and aggravating immunoparalysis (16). mtROS is a key signal for NET formation, so mitochondrial dysfunction alters NET quantity and quality (42); mtDNA within NETs further amplifies inflammation in a feed-forward loop (35). In sepsis-associated acute lung injury, neutrophil dysfunction drives excessive NETs and lung tissue injury (48, 49).

### 5.3 Parenchymal dysfunction in cardiomyocytes, renal tubular epithelium, and lung

Parenchymal mitochondrial dysfunction directly causes organ failure; cardiomyocytes and renal tubular epithelial cells have high energy demands and are especially vulnerable (40, 45). In sepsis-induced cardiomyopathy, mitochondria (>30% of cardiomyocyte volume) show cristae disruption, reduced respiratory-chain activity, increased mtROS, and abnormal opening of the mitochondrial permeability transition pore (mPTP), causing energy deficit, calcium disturbance, and cell death (32, 37, 40); lactate-mitochondria crosstalk and N6-methyladenosine modification of quality-control genes contribute (17, 21, 50). In sepsis-associated acute kidney injury, tubular cells depend on OXPHOS for reabsorption; sepsis causes bioenergetic failure, dynamic imbalance, and impaired mitophagy, with cardiolipin remodeling triggering mtDAMP release (3, 33, 41, 45). mtDAMPs from injured tubular cells activate immune cells, whose mediators in turn aggravate tubular mitochondrial damage (33, 41).

Acute lung injury and acute respiratory distress syndrome warrant comparable emphasis. Alveolar epithelial and endothelial cells, together with lung-recruited neutrophils and macrophages, sustain mitochondrial injury in which HIF-1α–driven glycolysis, excess mtROS, and NET-associated mtDNA promote barrier disruption and inflammatory infiltration (14, 48, 49). Comparing the three organs clarifies common versus specific pathways. Bioenergetic failure, mtROS overproduction, mtDNA/cGAS-STING and mtROS/NLRP3 activation, and cardiolipin remodeling are shared, convergent mechanisms. Organ-specific features differ by tissue: the heart is dominated by mPTP opening, calcium mishandling, and contractile energy failure; the kidney by tubular OXPHOS dependence and mitophagy imbalance; and the lung by alveolar-barrier disruption and NET-driven mtDNA amplification. These shared and specific axes are summarized in **Table 1**.

**Table 1:** Core mitochondrial immunometabolic mechanisms linking immune-cell dysfunction to organ failure in sepsis.

Mechanistic axis	Key mitochondrial/immunometabolic events	Main cellular context	Immune and pathophysiological consequences	Translational implication
Early hyperinflammatory reprogramming	Increased glycolysis; OXPHOS uncoupling; broken TCA cycle; succinate and itaconate; mtROS generation; HIF-1α/NF-κB activation.	Macrophages, neutrophils, and activated innate immune cells.	M1 polarization, proinflammatory cytokine production, NET formation, and systemic inflammatory amplification.	Phase-specific metabolic modulation (PDK-PDC and glycolysis-OXPHOS balance) rather than nonspecific immunosuppression.
Late bioenergetic failure and immunoparalysis	Sustained OXPHOS inhibition, ATP depletion, and reduced mitochondrial mass or respiratory capacity.	PBMCs, monocytes/macrophages, dendritic cells, and T cells.	Endotoxin tolerance, impaired antigen presentation, T-cell exhaustion, and vulnerability to secondary infection.	Respiratory profiling, circulating mtDNA, and immunometabolic endotyping to guide immune-restorative strategies.
Mitochondrial quality-control imbalance	Time- and cell-type-dependent mitophagy; PINK1-Parkin; Drp1/Fis1 fission; reduced Mfn1/2 and OPA1; PGC-1α dysregulation.	Macrophages, neutrophils, renal tubular epithelial cells, and cardiomyocytes.	Balanced control limits mtDAMP release; insufficient or excessive mitophagy and pathological fission promote dysfunction and injury.	Stage- and cell-specific restoration of adaptive turnover and network integrity.
mtDNA release and innate immune sensing	Cytoplasmic or extracellular mtDNA; activation of cGAS-STING and TLR9 signaling.	Dendritic cells, macrophages, neutrophils, and renal tubular epithelial cells.	Interferon/inflammatory signaling, impaired antigen presentation, and DAMP-driven systemic inflammation.	Target mtDNA release or downstream signaling; monitor cell-free mtDNA for risk stratification.
mtROS-NLRP3 inflammasome-pyroptosis axis	Excess mtROS; NLRP3 assembly; caspase-1 activation; IL-1β/IL-18 maturation; pyroptosis.	Macrophages, neutrophils, cardiomyocytes, and renal tubular epithelial cells.	Threshold-dependent signaling: moderate mtROS aids defense, whereas excess drives cytokine release and tissue injury.	Restore mtROS homeostasis (UCP2, glutathione set-point); BAM15/targeted antioxidants with careful patient selection.
Cardiolipin-centered lipid remodeling	Cardiolipin oxidation and externalization; impaired supercomplex stability; disrupted FAO and PPAR signaling.	Cardiomyocytes, renal tubular epithelial cells, hepatocytes, and macrophages.	Respiratory dysfunction, ATP deficit, increased mtDAMP release, and NLRP3 activation.	Membrane stabilization, FAO restoration, and PPAR-related, heterogeneity-sensitive interventions.
Immune-organ mitochondrial crosstalk	Bidirectional exchange of cytokines, mtDAMPs, NETs, and lactate between immune and parenchymal cells.	Immune cells with cardiomyocytes, renal tubular, and lung/gut/liver compartments.	Self-amplifying loops contributing to septic cardiomyopathy, SA-AKI, and acute lung injury/ARDS.	Mitochondrial transplantation, exosome delivery, substrate intervention, and multi-omics monitoring.
Gut–liver barrier axis and organ comparison	Intestinal-epithelial OXPHOS failure and barrier loss; Kupffer-cell/hepatocyte Drp1-mtDNA-STING; shared versus organ-specific parenchymal injury.	Intestinal epithelium, Kupffer cells, hepatocytes; heart, kidney, and lung parenchyma.	Endotoxin translocation and gut-liver amplification; common bioenergetic/mtDAMP mechanisms with organ-specific features (heart mPTP; kidney tubular mitophagy; lung barrier/NET-mtDNA).	Barrier protection, microbiota-metabolite modulation, and Kupffer-cell mtDNA-STING inhibition.

ARDS, acute respiratory distress syndrome; cGAS-STING, cyclic GMP-AMP synthase–stimulator of interferon genes; DAMPs, damage-associated molecular patterns; DCA, dichloroacetate; FAO, fatty acid oxidation; GSH, glutathione; mtDAMPs, mitochondrial DAMPs; mtDNA, mitochondrial DNA; mtROS, mitochondrial reactive oxygen species; NETs, neutrophil extracellular traps; NLRP3, NOD-like receptor family pyrin domain containing 3; OXPHOS, oxidative phosphorylation; PBMCs, peripheral blood mononuclear cells; PDC, pyruvate dehydrogenase complex; PDK, pyruvate dehydrogenase kinase; PPAR, peroxisome proliferator-activated receptor; SA-AKI, sepsis-associated acute kidney injury; TCA, tricarboxylic acid; TLR9, Toll-like receptor 9; UCP2, uncoupling protein 2.

### 5.4 The gut–liver mitochondrial immunometabolic axis

The gut and liver, key immune-barrier organs that are frequently overlooked, are integral to sepsis-associated mitochondrial immunometabolism. Intestinal epithelial cells are highly OXPHOS-dependent; septic mitochondrial dysfunction and mtROS impair their bioenergetics and tight-junction integrity, disrupting the epithelial barrier and permitting translocation of microbial products such as endotoxin into the portal and systemic circulation (51). These gut-derived signals damage mitochondria in distant organs and, delivered via the portal vein, converge on the liver. In hepatocytes and especially Kupffer cells, sepsis triggers respiratory-chain failure, excess mtROS, and Drp1-dependent fission that releases mtDNA and activates STING signaling, driving cytokine release and hepatic injury; the resulting DAMPs feed back into systemic inflammation (52). This gut–epithelium–liver–immune circuit constitutes a self-reinforcing mitochondrial axis and an emerging, tractable target: barrier protection, microbiota-derived metabolite modulation, and Kupffer-cell mtDNA-STING inhibition, for limiting multi-organ injury (51, 52).

## 6 Conclusions and perspectives

### 6.1 Current knowledge gaps: temporal dynamics, cellular heterogeneity, and clinical translation

Several gaps remain. First, temporal dynamics are poorly defined: the metabolic features of early and late sepsis differ, yet the stage-specific roles of quality-control mechanisms lack systematic study (8, 28). Second, cellular heterogeneity is incompletely resolved; immune subsets have distinct mitochondrial features, and the same cell type varies across tissue microenvironments (4, 7, 43); single-cell omics offers tools but remains nascent in sepsis. Third, the gap between preclinical findings and clinical translation is the greatest challenge, reflecting model-to-human differences, patient heterogeneity, and issues of drug specificity, bioavailability, and safety (6, 8, 15, 24); bedside mitochondrial monitoring also lacks standardization (24, 53).

### 6.2 Future directions: mitochondria-targeted therapy and precision immunometabolic monitoring

Future directions center on three areas. Mitochondria-targeted therapies are advancing, mitochondrial transplantation restores immune-cell metabolism and partially rescues the TCA cycle and nucleotide metabolism in septic rats (47), and exosome-mediated delivery (e.g., circRNA mSCAR) modulates macrophage polarization to attenuate inflammation (46). Metabolic substrate intervention, dichloroacetate activating PDC via PDK inhibition, and BAM15 lowering mtROS are protective in models but require more safety data (19, 20, 36). Precision immunometabolic monitoring, integrating metabolomics, proteomics, and single-cell transcriptomics with circulating mtDNA and respiratory-function testing as biomarkers, can stratify patients and advance sepsis care from empirical management toward precision medicine (10, 15, 35, 53, 54). Future studies should also clarify how persistent mitochondrial dysfunction contributes to post-sepsis syndrome (55) and determine whether and how biological sex modifies cardiac mitochondrial responses during sepsis (56).

### 6.3 Limitations

Several limitations of this review should be acknowledged. As a mini-review, its coverage is selective rather than systematic, and the constraints of the format preclude exhaustive treatment of every organ, cell type, and pathway; topics such as the gut–liver axis and lymphocyte subsets are introduced concisely rather than comprehensively. Much of the mechanistic evidence derives from rodent models and *in vitro* systems whose correspondence to human sepsis is imperfect, and the marked temporal, cellular, and patient-level heterogeneity of sepsis limits generalization. Finally, standardized methods for bedside assessment of human mitochondrial function are still lacking, so the translational proposals discussed here remain hypotheses that require prospective clinical validation. These limitations also define the priorities for future work.

In summary, mitochondrial immunometabolism provides an integrative framework for understanding immune dysfunction and organ failure in sepsis. Progress will require deeper study of temporal dynamics, cellular heterogeneity, and clinical translation to convert mechanistic insight into strategies that improve the prognosis of septic patients.

## Supplementary material

Supplementary Figure 1 Cross-regulation of mitochondrial damage-associated molecular pattern (mtDAMP)–sensing pathways in sepsis. Damaged or dysfunctional mitochondria simultaneously release mtDNA, mitochondrial reactive oxygen species (mtROS), and remodeled/externalized cardiolipin. These mtDAMPs engage distinct sensors: cytosolic mtDNA activates the cGAS–STING axis to induce type I interferon, whereas mtROS and externalized cardiolipin activate the NLRP3 inflammasome to drive IL-1β/IL-18 maturation and pyroptosis (canonical signaling, dark arrows). Critically, the pathways cross-regulate one another (crimson dashed arrows): mtROS promotes further mtDNA oxidation and release; oxidized mtDNA acts as a particularly potent NLRP3 ligand that links the cGAS–STING and inflammasome axes; externalized cardiolipin lowers the NLRP3 activation threshold; and type I interferon downstream of STING sensitizes cells to subsequent inflammasome activation. The result is a feed-forward, self-amplifying network in which each node both triggers and is triggered by the others, so that blockade of any single node is often insufficient. The network nodes therefore represent candidate targets for combination-based intervention.
